# Professional Training and Clinical/Radiographic Factors Associated With Endodontic Decision‐Making: A Before‐and‐After Study

**DOI:** 10.1111/eje.70125

**Published:** 2026-03-06

**Authors:** Luahra Peserico, Isabella M. Lena, Gabriela. S. Liedke, Raquel P. Antoniazzi, Roberta K. Scarparo, Renata D. Morgental

**Affiliations:** ^1^ Graduate Program in Dental Science Federal University of Santa Maria Santa Maria Rio Grande do Sul Brazil; ^2^ Department of Stomatology and Graduate Program in Dental Science Federal University of Santa Maria Santa Maria Rio Grande do Sul Brazil; ^3^ Department of Conservative Dentistry and Graduate Program in Dentistry Federal University of Rio Grande do Sul Porto Alegre Rio Grande do Sul Brazil

**Keywords:** clinical decision‐making, dental education, dental students, endodontics, retreatment

## Abstract

**Introduction:**

Endodontic decision‐making is influenced by clinicians' professional training and by the clinical and radiographic characteristics of each case. This study aimed to assess the association of these factors with treatment decisions and to determine whether the addition of clinical information modifies treatment choices compared with radiographic analysis alone.

**Materials and Methods:**

Thirty clinical cases involving endodontically treated teeth were evaluated through electronic questionnaires by 56 participants with different educational backgrounds (undergraduate students, general practitioners, postgraduate endodontic students, and endodontists). In the first questionnaire (Q1), only periapical radiographs were provided. One month later, a second questionnaire (Q2) included additional clinical information. Participants selected the most appropriate treatment option for each case. Changes in decisions between Q1 and Q2 (categorised as wait‐and‐see vs. reintervention) were analysed using the McNemar test. Associations between examiner‐ and tooth‐related factors and treatment decisions were assessed using multilevel binary logistic regression (*α* = 0.05).

**Results:**

Wait‐and‐see and nonsurgical retreatment were the preferred options at both time points. Endodontists recommended reintervention more frequently in Q2 than in Q1. In Q1, older examiners were more likely to recommend reintervention. Decisions were also influenced by tooth‐related factors, with altered periapical conditions and inadequate root canal fillings being associated with a higher likelihood of reintervention, whereas posterior teeth, iatrogenic errors, and inadequate coronal restorations/prosthetic crowns were associated with a lower likelihood. In Q2, specialty training in endodontics and prior reintervention decisions in Q1 were strong predictors of reintervention, along with selected clinical and radiographic factors.

**Conclusions:**

Conservative approaches were generally preferred. However, professional training and specific clinical and radiographic characteristics significantly influenced reintervention decisions.

## 1 Introduction

Apical periodontitis (AP) is traditionally defined as an inflammatory condition affecting the periapical tissues that results from microbial colonisation of the root canal system (RCS) [1]. Although endodontic treatment (ET) is effective in most cases, AP remains a common finding in previously treated teeth, with a reported prevalence of up to 39% [2], often requiring a new clinical decision regarding the need for reintervention. In such cases, different therapeutic approaches may be adopted, including no treatment, monitoring (wait‐and‐see), nonsurgical retreatment, surgical intervention, or extraction of the affected tooth [3].

Decision‐making in endodontics is influenced by both tooth‐related factors and practitioner‐related characteristics [4, 5, 6, 7, 8, 9, 10]. Educational level, clinical experience, and self‐confidence are key determinants of treatment choice, whereas radiographic and clinical variables, such as root filling quality, coronal restoration, periapical status, and time since treatment, also play decisive roles [4, 5, 6, 7, 8, 11, 12, 13, 14]. The interaction among these variables, combined with the absence of standardised guidelines, may contribute to diagnostic and therapeutic discrepancies among professionals.

Recent studies indicate that the way clinical information is presented and made available may further influence endodontic decision‐making [3, 15]. The inclusion of complementary data, such as the presence of signs and/or symptoms, restorative conditions, and time since treatment completion, can modify the clinician's perception of case complexity and prognosis. However, little is known about how the format of information presentation (radiographic analysis alone versus radiographic analysis combined with clinical data) affects therapeutic decisions, particularly across different educational levels.

Although comprehensive clinical assessment is essential for treatment planning in real‐world settings, studies based solely on radiographic evaluation provide valuable insight into the cognitive aspects of endodontic decision‐making. This approach allows assessment of how the availability of clinical information may influence diagnostic interpretation and therapeutic choices. Therefore, the present study aimed to evaluate the association between professional training and clinical and radiographic factors with endodontic decision‐making. Additionally, it investigated the impact of supplementary clinical information on the treatment options selected by students and professionals, compared with decisions based solely on radiographic analysis.

## 2 Materials and Methods

This study was approved by the Institutional Ethics Committee (Protocol No. 26387819.9.0000.5346). Informed consent was obtained from all participants prior to data collection.

### 2.1 Selection of Participants and Clinical Cases

The data analysed in this study were derived from a previous investigation [16]. The sample size was based on a previously published investigation addressing endodontic retreatment decision‐making [13], which served as a reference for sample definition, although no formal sample size calculation was performed.

A total of 56 participants with different levels of academic training and clinical experience (*n* = 14 per group). Participants were categorised into four groups: undergraduate students (US), general practitioners (GP), postgraduate endodontic students (PS), and endodontists (EN). The US group included final‐year dental students. The GP group consisted of general dentists without formal postgraduate training in endodontics, such as advanced courses or specialisation programs. The PS group comprised clinicians in the final year of their endodontic specialised training. Finally, the EN group was composed of certified endodontists who had completed a specialisation program with a minimum of 750 h of coursework.

Participants in the GP and EN groups were randomly selected from a list of professionals registered with the Regional Dental Council of Santa Maria (Santa Maria, RS, Brazil) and were contacted via email and/or telephone. A similar process was adopted for selecting participants in the US and PS groups, involving final‐year undergraduate students from the Faculty of Dentistry at Federal University of Santa Maria and students in the final year of the Endodontic Specialisation Program at Uningá Eleva, respectively.

The questionnaires were developed using 30 digital periapical radiographs obtained from the clinical records of patients treated at university dental clinics. Case selection followed predefined eligibility criteria, including radiographs with satisfactory image quality depicting a single endodontically treated tooth and fully encompassing the periapical region; images that did not meet these criteria were excluded. Additional information on the questionnaire development, including the complete list of cases and their respective clinical and radiographic characteristics, is provided in Table S1. The selected cases represented a wide range of clinical scenarios commonly encountered in dental practice and potentially influencing endodontic decision‐making.

### 2.2 Questionnaire Structure and Radiographic Interpretation

Each participant received two questionnaires (Q1 and Q2) administered 1 month apart. The questionnaires were developed using an online survey platform (Google Forms; Google LLC, Mountain View, CA, USA) and distributed electronically. Responses were collected between March 2020 and May 2021. Participants were free to choose the electronic device most convenient for completing the assessment. The only instruction provided was to interpret the radiographic images in a dimly lit environment to minimise external interference and enhance image visualisation.

In the first questionnaire (Q1), participants provided demographic information (age and sex) and details regarding their professional training (level of endodontic training and years of clinical experience). They then evaluated 30 periapical radiographs without access to additional information. The use of radiographic images alone in Q1 was intentional and aimed to isolate the perceptual and cognitive aspects of decision‐making, thereby minimising potential confounding factors related to the availability of clinical information.

In the second questionnaire (Q2), participants reassessed the same radiographic images, this time with access to complementary clinical information, including medical and dental records, as illustrated in Figure S1. Decisions were primarily based on radiographic assessment, supported by brief clinical information, specifically time since completion of endodontic treatment, presence of signs and/or symptoms, and quality of coronal restoration/prosthetic crown. No clinical photographs or direct examinations were used, and prosthetic discrepancies were not systematically assessed. To minimise memory bias and prevent recall of the responses provided in the first questionnaire, the sequence of clinical cases was randomised before administration of Q2.

In both questionnaires, participants were instructed to select the most appropriate treatment option for each endodontically treated tooth. The available options included: wait‐and‐see, nonsurgical retreatment, apical surgery, nonsurgical retreatment and apical surgery, or extraction.

### 2.3 Clinical Case Categorization

The categorization of clinical cases was performed by a panel of experienced evaluators consisting of three endodontists and one radiologist. The classification process was conducted until consensus was reached, thereby establishing a gold standard for the analysis of clinical and radiographic factors. The categorization criteria adopted were as follows:

- Dental group: (i) anterior or (ii) posterior.
- Dental arch: (i) maxilla or (ii) mandible
- Time since the end of ET: (i) up to 1 year, (ii) 2–5 years, or (iii) 6 years or more [17].
- Presence of intracanal post and/or prosthetic crown: (i) yes or (ii) no
- Presence of iatrogenic errors: (i) yes, including canal transportation, root perforation, or instrument fracture; or (ii) no.
- Presence of signs and/or symptoms: (i) yes, with manifestation such as fistulous tract, swelling, or pain; or (ii) no [18].
- Root filling quality (density): (i) adequate, characterised by uniform radiodensity and complete adaptation of the material to the canal walls; or (ii) inadequate, defined by the presence of visible lateral voids along the filling, internal gaps within the obturation material, or identification of untreated canals [19].
- Root filling quality (length): (i) short, when the filling material terminates more than 2 mm short of the radiographic apex; (ii) adequate, when it extends between 0 and 2 mm short of the apex; or (iii) overextended, when there is extrusion of the material beyond the apex [19]. For analytical purposes, this variable was later dichotomized, classifying categories (i) and (iii) as inadequate root canal filling length
- Periapical status: (i) normal periodontal ligament, (ii) widened periodontal ligament or (iii) periapical lesion [20]. For statistical analysis, this variable was dichotomized, grouping category (i) as healthy and categories (ii) and (iii) as diseased.
- Quality of coronal restoration/prosthetic crown: (i) adequate, defined by the radiographic integrity of the restoration; or (ii) inadequate, characterised by the absence of restoration or the presence of detectable signs of open margins, overhangs, or secondary caries [19].

In the assessment of root filling quality (length and density) and periapical status in multirooted teeth, the root presenting the worst‐case scenario was considered. The main characteristics of the selected cases are presented in Table 1.

**TABLE 1:** Characteristics of the selected clinical cases (*n* = 30).

Characteristics of the clinical cases	*N* (%)
Dental group	
Anterior	8 (26.7)
Posterior	22 (73.3)
Dental arch	
Mandible	15 (50)
Maxilla	15 (50)
Post and/or prosthetic crown	8 (26.7)
Time since the end of ET	
≤ 1 year	6 (20)
2–5 years	13 (43.3)
≥ 6 years	11 (36.7)
Iatrogenic errors	3 (10)
Signs and/or symptoms	12 (40)
Inadequate root filling quality (density)	5 (16.7)
Inadequate root filling quality (length)	12 (40)
Periapical disease	17 (56.7)
Inadequate coronal restoration/prosthetic crown	12 (40)

Abbreviation: ET, endodontic treatment.

### 2.4 Statistical Analysis

Data analysis was conducted using SPSS version 26.0 (SPSS Inc., Chicago, IL, USA). Changes in the frequency of treatment options from Q1 to Q2 were assessed using the McNemar test. For this analysis, the outcome was categorised into two groups: wait‐and‐see and reintervention. The latter category included the treatment options nonsurgical retreatment, apical surgery, nonsurgical retreatment, and apical surgery, and extraction.

Multilevel binary logistic regression models were used to assess the influence of different factors on the decision‐making process. The following examiner‐related independent variables were considered: age (dichotomized at the median ≤ 29 vs. > 29 years), gender (female or male), and professional training (US, GP, PS, or EN). In addition, the following independent variables were investigated: dental group; dental arch; post and/or prosthetic crown; time since the completion of ET; iatrogenic errors; signs and/or symptoms; root filling quality (dichotomized as adequate or inadequate, based on length and density); periapical status; and quality of coronal restoration/prosthetic crown.

As dependent variables, clinical decision‐making (wait‐and‐see vs. reintervention) in Q1 and Q2 were considered. Additionally, the treatment option selected in Q1 was included as an independent variable when the dependent variable was the treatment option in Q2. The analysis structure included fixed effect with a random intercept. The multilevel structure considered individuals (level 1) and clinical (level 2). Model 1 included individual‐level variables, and Model 2 consisted of Model 1 plus level‐2 variables (“full model”). Variables with a *p‐value* < 0.05 in the unadjusted analyses were entered into the adjusted models as potential confounders.

## 3 Results

Table 2 shows the baseline characteristics of the 56 participants (*n* = 14/group). Table 3 shows the distribution of treatment options selected by each group at both time points (Q1 and Q2). At both time points, the wait‐and‐see option was the most frequently selected by participants, whereas extraction was the least frequently chosen alternative. No significant differences among groups were observed in Q1 (*p =* 0.075); however, in Q2, treatment option distribution differed significantly among groups (*p* < 0.001). In within‐group comparisons (Figure 1), the EN group was observed to recommend reintervention more frequently in Q2 than in Q1 (*p* = 0.008), indicating a change in decision‐making patterns over time. In contrast, the other groups maintained similar decision patterns between the two assessments (US, *p* = 0.273; GP, *p* = 0.759; PS, *p* = 0.165).

**TABLE 2:** Baseline characteristics of the participants.

Variable	US	GP	PS	EN	*p*
Gender [*N* (%)]					0.304
Female	11 (78.6)	7 (50.0)	11 (78.6)	10 (71.4)	
Male	3 (21.4)	7 (50.0)	3 (21.4)	4 (28.6)	
Age	23.6 ± 1.3	42.3 ± 12.4	26.4 ± 4.1	40.2 ± 9.2	< 0.001^b^
Categorised age [*N* (%)]					< 0.001^a^
≤ 29 years	14 (100)	1 (7.1)	11 (78.6)	3 (21.4)	
> 29	0 (0)	13 (92.9)	3 (21.4)	11 (78.6)	
Experience [Mean (±SD)]	0 ± 0	19.3 ± 11.2	5.2 ± 6.7	18.8 ± 8.7	< 0.001^b^
Experience in endodontics [Mean (±SD)]	—	—	—	14.36 (5.82)	—

*Note:* Age and experience are expressed in years.

Abbreviations: EN, endodontists; GP, general practitioners; PS, postgraduate endodonticendodontics students; SD, standard deviation; US, undergraduate students.

^a^ Chi‐square test.

^b^ Kruskal–Wallis test.

**TABLE 3:** Frequency distribution of treatment options preferred by the four groups of participants at each moment of the study.

	Treatment	US	GP	PS	EN	*p* ^a^
Q1 Isolated radiographic analysis *N* (%)	Wait‐and‐see	260 (61.9)	259 (61.7)	269 (64.0)	252 (60.0)	0.075
	NSR	135 (32.1)	130 (31.0)	138 (32.9)	157 (37.4)	
	Apical surgery	6 (1.4)	10 (2.4)	4 (1.0)	4 (1.0)	
	NSR and apical surgery	13 (3.1)	17 (4.0)	7 (1.7)	6 (1.4)	
	Extraction	6 (1.4)	4 (1.0)	2 (0.5)	1 (0.2)	
	Total	420 (100)	420 (100)	420 (100)	420 (100)	
Q2 Radiographic analysis and clinical history *N* (%)	Wait‐and‐see	243 (57.9)	263 (62.6)	252 (60.0)	223 (53.1)	< 0.001
	NSR	143 (34.0)	131 (31.2)	161 (38.3)	187 (44.5)	
	Apical surgery	9 (2.1)	8 (1.9)	1 (0.2)	3 (0.7)	
	NSR and apical surgery	24 (5.7)	13 (3.1)	6 (1.4)	5 (1.2)	
	Extraction	1 (0.2)	5 (1.2)	0 (0)	2 (0.5)	
	Total	420 (100)	420 (100)	420 (100)	420 (100)	

Abbreviations: EN, endodontists; GP, general practitioners; NSR, nonsurgical retreatment; PS, postgraduate endodontic students; US, undergraduate students.

^a^ Chi‐square test.

**FIGURE 1:**
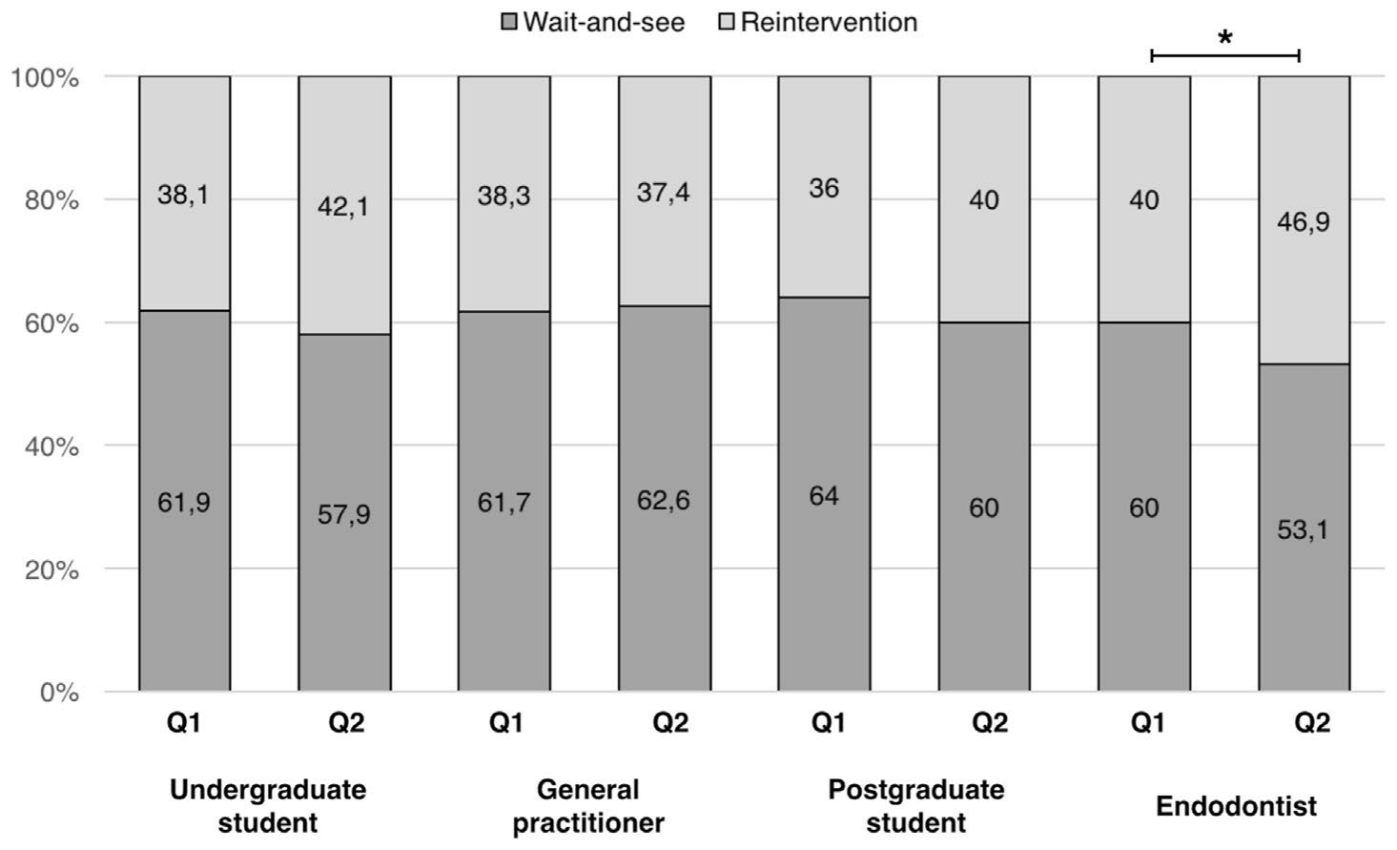
Changes in the frequencies of treatment options from Q1 (isolated radiographic analysis) to Q2 (radiographic analysis and clinical history) for each participants group. *p < 0.05, according to the McNemar test.

The multilevel regression models, in both the crude and adjusted analyses, for clinical decision‐making at Q1 and Q2, are presented in Tables 4 and 5, respectively. In Q1, the examiner's age was the only examiner‐related variable significantly associated with the outcome, with examiners > 29 years old being more likely to recommend reintervention compared to those ≤ 29 years old (IC 95%: 1.04–1.56). In the adjusted analysis, several clinical variables demonstrated a significant influence on the decision to recommend reintervention, including dental group, iatrogenic errors, root filling quality, periapical status, and the quality of coronal restoration/prosthetic crown. Posterior teeth were associated with a lower likelihood of reintervention recommendation compared with anterior teeth (95% CI: 0.30–0.53), as was the presence of iatrogenic errors (95% CI: 0.45–0.96). In addition, teeth with inadequate root canal filling were 2.69 times more likely to be recommended for reintervention compared with those with adequate filling (95% CI: 2.09–3.46). Teeth presenting altered periapical conditions showed a higher likelihood of reintervention recommendation (95% CI: 3.86–6.42), whereas teeth with inadequate coronal restoration or prosthetic crown exhibited a lower likelihood of reintervention recommendation (95% CI: 0.43–0.68). Although the dental arch was associated with the outcome in the crude analysis, it did not retain statistical significance after adjusting for other variables.

**TABLE 4:** Unadjusted and adjusted multilevel binary logistic regression analyses of the association between individual and clinical variables and the decision to reintervene in Q1.

Variables	Unadjusted	Model 1	Model 2
	OR (95% CI)		
Individual (*N* = 56)			
Age			
≤ 29 years	1.0 (Ref)	1.0 (Ref)	
> 29	1.27 (1.04–1.56)*	1.27 (1.04–1.56)*	
Gender		—	
Male	1.0 (Ref)		
Female	1.07 (0.84–1.35)		
Professional training		—	
General practitioners	1.0 (Ref)		
Endodontists	1.07 (0.81–1.42)		
Undergraduate students	0.99 (0.75–1.31)		
Postgraduate endodontic students	0.90 (0.68–1.20)		
Clinical (*N* = 1680)			
Dental Group			
Anterior	1.0 (Ref)		1.0 (Ref)
Posterior	0.72 (0.58–0.90)*		0.39 (0.30–0.53)**
Dental arch			
Mandible	1.0 (Ref)		1.0 (Ref)
Maxilla	1.29 (1.06–1.57)*		0.79 (0.61–1.03)
Post and/or prosthetic crown			—
No	1.0 (Ref)		
Yes	0.94 (0.75–1.18)		
Iatrogenic errors			
No	1.0 (Ref)		1.0 (Ref)
Yes	1.77 (1.29–2.45)**		0.66 (0.45–0.96)*
Root filling quality			
Adequate	1.0 (Ref)		1.0 (Ref)
Inadequate	2.60 (2.13–3.19)**		2.69 (2.09–3.46)**
Periapical status			
Healthy	1.0 (Ref)		1.0 (Ref)
Diseased	4.65 (3.72–5.83)**		4.98 (3.86–6.42)**
Quality of coronal restoration/prosthetic crown			
Adequate	1.0 (Ref)		1.0 (Ref)
Inadequate	0.60 (0.49–0.74)**		0.54 (0.43–0.68)**

*Note:* Model 1 (“individual model”): individual covariates; Model 2 (“full model”): clinical‐ and individual‐level covariates.

Abbreviations: CI, confidence interval; OR, odds ratio; Q1, first questionnaire (isolated radiographic analysis); Ref, reference.

*p‐value < 0.05; **p‐value < 0.01.

**TABLE 5:** Unadjusted and adjusted multilevel binary logistic regression analyses of the association between individual and clinical variables and the decision to reintervene in Q2.

Variables	Unadjusted	Model 1	Model 2
	OR (95% CI)		
Individual (*N* = 56)			
Age			
≤ 29 years	1.0 (Ref)	—	
> 29	0.98 (0.80–1.19)		
Gender			
Male	1.0 (Ref)	—	
Female	0.90 (0.72–1.12)		
Professional training			
General practitioners	1.0 (Ref)	1.0 (Ref)	
Endodontists	1.48 (1.12–1.95)*	1.51 (1.13–2.02)*	
Undergraduate students	1.22 (0.93–1.61)	1.25 (0.94–1.68)	
Postgraduate endodontic students	1.12 (0.85–1.48)	1.17 (0.87–1.57)	
Treatment option in Q1			
Wait‐and‐see	1.0 (Ref)	1.0 (Ref)	
Reintervention	3.82 (3.10–4.70)**	3.83 (3.11–4.71)**	
Clinical (*N* = 1680)			
Dental Group			—
Anterior	1.0 (Ref)		
Posterior	1.18 (0.95–1.48)		
Dental arch			
Mandible	1.0 (Ref)		1.0 (Ref)
Maxilla	1.39 (1.14–1.69)**		1.57 (1.20–2.04)*
Time since the end of ET			
≤ 1 years	1.0 (Ref)		1.0 (Ref)
2–5 years	0.74 (0.57–0.96)*		1.52 (1.10–2.10)*
≥ 6 years	0.62 (0.47–0.81)*		1.40 (0.99–1.99)
Post and/or prosthetic crown			
No	1.0 (Ref)		1.0 (Ref)
Yes	2.82 (2.21–3.61)**		1.38 (1.02–1.87)*
Iatrogenic errors			
No	1.0 (Ref)		1.0 (Ref)
Yes	1.62 (1.18–2.23)*		1.83 (1.19–2.81)*
Signs and/or symptoms			
No	1.0 (Ref)		1.0 (Ref)
Yes	7.26 (5.83–9.04)**		7.77 (5.81–10.40)**
Root filling quality			
Adequate	1.0 (Ref)		1.0 (Ref)
Inadequate	1.63 (1.34–1.99)**		1.46 (1.08–1.99)*
Periapical status			
Healthy	1.0 (Ref)		
Diseased	2.58 (2.10–3.16)**		0.96 (0.71–1.28)
Quality of coronal restoration/prosthetic crown			
Adequate	1.0 (Ref)		1.0 (Ref)
Inadequate	2.38 (1.94–2.90)**		0.92 (0.71–1.21)

*Note:* Model 1 (“individual model”): adjusted for individual covariates; Model 2 (“full model”): adjusted for clinical‐ and individual‐level covariates.

Abbreviations: CI, confidence interval; OR, odds ratio; Q2, second questionnaire (radiographic analysis and clinical history); Ref, reference.

*p‐value < 0.05; **p‐value < 0.001.

In Q2, in the adjusted analysis, several clinical variables were significantly associated with the decision to recommend reintervention. Teeth located in the maxilla showed a higher likelihood of reintervention recommendation compared with mandibular teeth (95% CI: 1.20–2.04). Regarding the time elapsed since the end of endodontic treatment, teeth treated between two and 5 years previously were more likely to be recommended for reintervention than those treated 1 year or less earlier (95% CI: 1.10–2.10). The presence of a post and/or prosthetic crown (95% CI: 1.02–1.87), as well as the occurrence of iatrogenic errors (95% CI: 1.19–2.81), was associated with a higher likelihood of reintervention recommendation. Cases presenting clinical signs and/or symptoms showed a substantially higher likelihood of reintervention recommendation (OR = 7.77; 95% CI: 5.81–10.40), as did teeth with inadequate root canal filling (95% CI: 1.08–1.99). Periapical status and the quality of coronal restoration/prosthetic crown, although statistically associated with the outcome in the crude analysis, did not retain significance in the adjusted analysis.

## 4 Discussion

The clinical management of teeth previously subjected to ET has been widely investigated and remains a topic of ongoing debate. Reaching a consensus on decision‐making among students and professionals with different levels of endodontic training remains challenging. This process is influenced by a wide range of factors, both related to the practitioner and the characteristics of the tooth [6, 21], as confirmed by the findings of the present study.

Participants selected the most appropriate treatment option from five alternatives, as described in previous studies [7]. At both assessment points (Q1 and Q2), students and professionals favoured the wait and see and nonsurgical retreatment options, a finding consistent with earlier research [6, 7, 15]. This preference may be related to the lower complexity of the analysed cases, as 18 out of 30 were asymptomatic and 13 out of 30 showed no evident periapical pathology (Table 1). Nonsurgical retreatment, with reported success rates of up to 93% [22] is typically prioritised over more invasive interventions. This same factor may explain the low frequency of “extractions”, which were selected in less than 2% of cases, reinforcing the preference for conservative approaches whenever the prognosis is favourable.

Changes in the frequencies of treatment options between Q1 and Q2 were also analysed. It was observed that endodontists recommended reintervention more frequently in Q2, when clinical information supplemented the radiographic images. This finding corroborates previous studies [4], suggesting that when relying solely on initial radiographic analysis, professionals may opt for a conservative approach, such as wait‐and‐see, and later reassess their decision. In this context, several investigations have analysed the influence of clinical signs and symptoms on endodontic decision‐making [6, 7, 13] yielding inconsistent results. According to Heinikainen et al. [13], general practitioners tend to underestimate the importance of patient‐reported symptoms and base their decisions predominantly on radiographic findings, which could explain the lack of significant variation in responses within this group between Q1 and Q2.

This study adopted a multilevel approach for data analysis, differing from most investigations on decision‐making in endodontics, which often use less complex statistical tests, such as the chi‐square test (*χ* ^2^), that assume independence between observations [6]. However, this assumption can compromise the validity of statistical inference and lead to the misidentification of associations, as it does not account for the existing dependence among data points [23]. In this study, intra‐individual correlations were incorporated, recognizing that each participant provided multiple responses throughout the evaluation of clinical cases. Therefore, the multilevel approach enables a more precise estimation of the effects of clinical and radiographic factors, adjusting for other variables related to both the clinical case and the professional's profile.

In Q1, age was the only examiner‐related variable significantly associated with the outcome in the adjusted analysis. Professionals over the age of 29 were more likely to recommend reintervention (OR: 1.27; 95% CI: 1.04–1.56). This finding is consistent with the study by Aryanpour et al. [24], which demonstrated an inverse relationship between practitioner age and the selection of the wait‐and‐see option in asymptomatic cases. However, this comparison should be interpreted cautiously, as the present study's decisions in Q1 were based solely on radiographic images, whereas Aryanpour et al. evaluated clinical scenarios supported by comprehensive patient information. Therefore, the similarity refers to the general influence of examiner experience rather than to a direct methodological equivalence. The time since graduation (years of experience) was also recorded as a relevant variable in this study, as it could provide an additional measure to assess the influence of professional training on decision‐making. However, due to its high collinearity with participants' age, this variable was removed from the analytical models to avoid statistical redundancy. In Q2, professional training was the primary factor influencing decision‐making. Endodontists were more likely to recommend reintervention compared with general practitioners (OR: 1.51; 95% CI: 1.13–2.02), reflecting their greater diagnostic expertise and specialised training [6]. These findings reinforce the impact of advanced education on the confidence and accuracy of clinical decisions, aligning with previous studies [4, 11, 15].

In Q1, the tooth group influenced the decision to recommend reintervention, with a lower probability of recommending reintervention for posterior teeth compared with anterior teeth (OR: 0.39; 95% CI: 0.30–0.53), possibly due to their greater anatomical and technical complexity [25, 26]. In Q2, the dental arch emerged as a determining factor, with a higher likelihood of reintervention in maxillary teeth (OR: 1.57; 95% CI: 1.20–2.04). The association of the dental arch with decision‐making observed only in Q2 may reflect an interaction between anatomical complexity and the additional clinical information provided in this stage. Factors such as the presence of symptoms, the time elapsed since endodontic treatment, and the existence of posts or restorations may have modified clinicians' perception of case difficulty and prognosis, leading to a higher tendency to recommend reintervention for maxillary teeth.

In Q2, the probability of recommending reintervention was significantly higher between 2 and 5 years after ET completion (OR: 1.52; 95% CI: 1.10–2.10), aligning with studies suggesting a more conservative approach within the first year, allowing for a longer observation period before intervening [5]. According to the European Society of Endodontology [17], when a 1‐year follow‐up radiograph shows a stable periapical lesion or only minimal reduction, the prognosis remains uncertain. In this scenario, a prolonged monitoring period is recommended before deciding on reintervention [27].

Another key factor in clinical decision‐making is the access to the RCS. The presence of an intracanal post and/or prosthetic crown limits coronal access and introduces additional risks during removal, such as excessive loss of dental structure, perforation, and root fracture [14, 28]. In the present study, these factors increased the likelihood of reintervention in Q2 (OR: 1.38; 95% CI: 1.02–1.87). Although the specific modality of reintervention was not assessed, prior studies indicate that, in such cases, clinicians tend to opt for more invasive approaches, such as apical surgery or extraction [14]. The presence of iatrogenic errors, such as fractured instruments, has already been mentioned as a critical factor that can influence decision‐making [6]. Alim‐Uysal et al. [11] reported that, in the absence of periapical pathology, 88.1% of endodontists opted for a wait‐and‐see approach, a proportion that dropped to 31.7% and 1.6% for lesions measuring 1 mm and 5 mm, respectively.

In the present study, iatrogenic errors reduced the likelihood of reintervention in Q1 (OR: 0.66; 95% CI: 0.45–0.96) but increased it in Q2 (OR: 1.83; 95% CI: 1.19–2.81), indicating that clinical information alters professional perception. Since teeth with intraoperative complications have lower success rates [26], reintervention often becomes unavoidable. Clinical signs and symptoms indicate an unfavourable prognosis and a greater need for reintervention [17], significantly influencing the choice of retreatment, surgery, or extraction (OR: 7.77; 95% CI: 5.81–10.40), as observed in previous studies [7, 24]. Similarly, root filling quality was associated with the outcome in Q1 (OR: 2.69; 95% CI: 2.09–3.46) and Q2 (OR: 1.46; 95% CI: 1.08–1.99), reinforcing that inadequate fillings increase the risk of ET failure [15, 21, 29, 30].

Periapical disease significantly influenced the recommendation for reintervention in Q1 (OR: 4.98; 95% CI: 3.86–6.42) but not in Q2. Studies indicate that AP increases the likelihood of more invasive treatments, particularly in extensive lesions [11]. In the present study, periapical status was dichotomized (healthy vs. diseased) according to the classification by Ng et al. [20], facilitating the analysis and replacing the periapical index (PAI), which is commonly applied in epidemiological studies [31]. In Q2, the presence of clinical signs and/or symptoms may have reduced the impact of periapical status and root filling quality, becoming the primary determinant of reintervention [7, 24]. While the quality of coronal restoration/prosthetic crown is known to influence the success of ET [32], its association with reintervention was significant only in Q1 (OR: 0.54; 95% CI: 0.43–0.68), suggesting a possible underestimation of its importance. This limitation may be related to the reliance on radiographic analysis, which does not always detect microleakage, perforations, and cracks [33].

This study was conducted on a limited sample from a specific region, which may influence participants' perceptions and interpretations. Despite this limitation, the findings support factors previously identified in studies conducted in other settings. Another potential limitation is the lack of standardisation of screens for radiographic evaluation, which is inherent to studies using electronic questionnaires. However, prior research has shown that the type of device used does not compromise diagnostic ability in cases such as root fractures [34] and internal root resorption [35]. Additionally, the absence of an option such as “unable to decide due to insufficient information” represents another limitation, as real clinical decision‐making also depends on data obtained from patient history and physical examination. Nevertheless, this methodological choice was intentional, aiming to specifically assess the influence of radiographic information—alone or in combination with basic clinical data—on endodontic decision‐making. The use of predefined response options allowed standardisation of answers and minimised interpretative variability among participants.

Clinical decision‐making in endodontics is inherently multifactorial and may be affected by cognitive biases, professional training, and clinical experience [36]. The lack of consensus among professionals, although expected in highly complex cases, highlights the need for greater uniformity in diagnostic reasoning. To address these disparities, dental curricula and continuing education programs should emphasise evidence‐based guidelines, standardised diagnostic criteria, and reflective clinical reasoning to enhance the consistency and accuracy of endodontic decisions.

## 5 Conclusion

Endodontic decision‐making was influenced by both professional training and clinical–radiographic factors, underscoring the complexity of retreatment choices in endodontics. Although conservative approaches were generally preferred, endodontists tended to indicate more reinterventions when additional clinical information was available, emphasising the importance of integrating clinical and radiographic findings. These findings highlight the need for clearer, evidence‐based clinical guidelines to support consistent decision‐making across different educational and clinical settings. Future studies should focus on evaluating educational interventions and decision‐support strategies aimed at improving diagnostic accuracy and treatment planning in endodontics.

## Author Contributions

All authors contributed to the study conception and design. Material preparation and data collection were performed by Luahra Peserico and Isabella Marian Lena. Data analysis was performed by Raquel Pippi Antoniazzi. The first draft of the manuscript was written by Luahra Peserico and all authors commented on previous versions of the manuscript. All authors read and approved the final manuscript.

## Funding

This study was partly funded by the “Coordination for the Improvement of Higher Education Personnel” (CAPES, Brazil), which supported the Master's scholarship for L.P. (Protocol n° 88887.648845/2021‐00).

## Conflicts of Interest

The authors declare no conflicts of interest.

## Supporting information


**Figure S1:** Radiographic image and clinical history provided in the second questionnaire (Q2). ET, Endodontic treatment.

**Table S1:** Characteristics of the selected clinical cases.

## Data Availability

The data that support the findings of this study are available from the corresponding author upon reasonable request.
