# Supplementary material for: Professional Training and Clinical/Radiographic Factors Associated With Endodontic Decision‐Making: A Before‐and‐After Study
Source: Eur J Dent Educ. 2026 Mar 6;30(4):1661–71. doi: 10.1111/eje.70125 (PMC13686686; doi:10.1111/eje.70125)
Supplement: Supplementary file 1 [file EJE-30-1661-s001.docx]

**Supplemental Table 1** Characteristics of the selected clinical cases.

| **Clinical/radiographic factors** | **1** | **2** | **3** | **4** | **5** | **6** | **7** | **8** | **9** | **10** | **11** | **12** | **13** | **14** | **15** | **16** | **17** | **18** | **19** | **20** | **21** | **22** | **23** | **24** | **25** | **26** | **27** | **28** | **29** | **30** |
| --- | --- | --- | --- | --- | --- | --- | --- | --- | --- | --- | --- | --- | --- | --- | --- | --- | --- | --- | --- | --- | --- | --- | --- | --- | --- | --- | --- | --- | --- | --- |
| Dental group |  |  |  | X | X |  |  | X |  |  |  |  |  | X |  |  |  | X |  |  |  | X | X |  |  | X |  |  |  |  |
| Dental arch |  | X | X |  |  | X |  |  | X | X |  |  |  | X | X |  |  |  |  | X | X |  |  | X | X |  | X | X | X | X |
| Time since the end of ET | 24 | 96 | 156 | 120 | 72 | 60 | 120 | 24 | 240 | 4 | 120 | 48 | 240 | 60 | 24 | 168 | 12 | 240 | 24 | 8 | 60 | 36 | 48 | 12 | 40 | 8 | 24 | 60 | 3 | 24 |
| Post and/or prosthetic crown | X |  |  |  |  |  |  |  |  |  | X |  |  |  | X |  |  | X | X |  |  |  | X |  |  |  | X |  | X |  |
| Iatrogenic errors |  |  |  |  |  |  |  |  |  |  |  |  |  |  |  | X |  |  |  |  | X |  |  |  |  |  |  |  | X |  |
| Signs and/or symptoms | X |  | X |  | X |  | X |  | X | X | X |  | X |  |  |  |  |  |  |  | X |  | X |  | X |  |  | X |  |  |
| Root filling quality (density) |  |  | X |  |  |  |  |  |  |  |  |  |  | X |  |  |  |  |  |  |  |  | X |  |  |  |  |  | X |  |
| Root filling quality (length) |  |  |  |  |  |  |  |  |  |  | X |  |  | X | X | X | X |  | X | X | X |  | X |  |  |  | X | X | X |  |
| Periapical status |  | X | X |  | X |  | X | X |  |  | X | X | X |  |  | X | X |  |  |  | X | X | X |  | X |  | X | X | X |  |
| Quality of coronal restoration/prosthetic crown | X |  | X | X |  |  |  |  | X | X | X | X |  |  |  |  | X |  |  |  | X |  | X |  | X | X |  |  |  |  |

Abbreviation: ET: Endodontic treatment. X mark was used for the following characteristics: dental group (anterior); dental arch (mandible); post and/or prosthetic crown (yes); iatrogenic errors (yes); signs and/or symptoms (yes); root filling quality regarding density and length (inadequate); periapical status (diseased); quality of coronal restoration/prosthetic crown (inadequate). For the time since the end of ET, data is expressed in months.
